# Transport Property of Wrinkled Graphene Nanoribbon Tuned by Spin-Polarized Gate Made of Vanadium-Benzene Nanowire

**DOI:** 10.3390/nano13152270

**Published:** 2023-08-07

**Authors:** Hong Yu, Yan Shang, Yangyang Hu, Lei Pei, Guiling Zhang

**Affiliations:** School of Materials Science and Chemical Engineering, Harbin University of Science and Technology, Harbin 150080, China; yuhong19881015@126.com (H.Y.); huyy@hrbust.edu.cn (Y.H.); plxinxiang@163.com (L.P.)

**Keywords:** spin-polarized, four-terminal device, wrinkled graphene nanoribbon, V_7_(Bz)_8_

## Abstract

A series of four-terminal V_7_(Bz)_8_-WGNR devices were established with wrinkled graphene nanoribbon (WGNR) and vanadium-benzene nanowire (V_7_(Bz)_8_). The spin-polarized V_7_(Bz)_8_ as the gate channel was placed crossing the plane, the concave (endo-positioned) and the convex (endo-positioned) surface of WGNR with different curvatures via Van der Waals interaction. The density functional theory (DFT) and nonequilibrium Green’s function (NEGF) methods were adopted to calculate the transport properties of these devices at various bias voltages (*V*_S_) and gate voltages (*V*_G_), such as the conductance, spin-polarized currents, transmission spectra (TS), local density of states (LDOS), and scattering states. The results indicate that the position of V_7_(Bz)_8_ and the bending curvature of WGNR play important roles in tuning the transport properties of these four-terminal devices. A spin-polarized transport property is induced for these four-terminal devices by the spin-polarized nature of V_7_(Bz)_8_. Particularly, the down-spin channel disturbs strongly on the source-to-drain conductance of WGNR when V_7_(Bz)_8_ is endo-positioned crossing the WGNR. Our findings on the novel property of four-terminal V_7_(Bz)_8_-WGNR devices provide useful guidelines for achieving flexible graphene-based electronic nanodevices by attaching other similar multidecker metal-arene nanowires.

## 1. Introduction

With the rapid development of the semiconductor industry, the miniaturization and integration of electronic devices have become an important research direction to adapt to the needs of the industry development. Low-dimensional materials have been proved to be the most promising materials for designing next-generation devices owing to their unique tunable electronic, optical, and mechanical properties [1,2,3]. Particularly, the fabrication of multi-terminal devices by using low-dimensional materials has attracted extensive interest [4,5,6].

Van der Waals heterostructures (vdWHs) composed of low-dimension materials have become a hot topic for scientific research, especially when their unusual manifestations display unexpected integrated properties [7,8,9]. So far, numerous researchers have strongly demonstrated that vdWHs based on graphene are the most promising materials for potential applications in electronic and optoelectronic fields. Generally, graphene could form various vdWHs with zero-dimensional (0D) quantum dots and plasmonic nanoparticles such as Ag nanoparticles [10,11], one-dimensional (1D) nanoribbons and nanowires such as CdS nanowires, carbon nitride sheet [12,13], as well as two-dimensional (2D) nanomaterials such as hexagonal boron nitride, layered transition metal dichalcogenides and metal oxides [14,15,16,17,18,19,20,21], referred to as 0D-G, 1D-G, and 2D-G vdWHs, respectively. Especially, the 1D-G vdWHs have already been used to fabricate many transistors with high speed and flexibility by using the 1D structure as a gate [22,23]. For instance, the scaled on-current and the transconductance of the transistors constructed using a Co_2_Si/Al_2_O_3_ core/shell nanowire aligned on top of the graphene as the gate can reach 3.32 mA·μm^−1^ and 1.27 mS·μm^−1^, respectively [22]. Using Al_2_O_3_ nanoribbons as the top gate, the 1D-G vdWHs transistors have been built up to exhibit superior performance, with the highest carrier mobility up to 23,600 cm^2^/V·s [23]. And thus, the 1D gate material plays a significant role in governing the transistor behavior. Despite these extraordinary achievements, designing new 1D gate materials to manipulate desirable signatures of graphene vdWHs is still in the initial stage and has great prospects. Particularly, the experimental synthesis and theoretical investigation of graphene vdWHs with a magnetic 1D gate material have so far remained elusive. 

Governed by the supernal flexibility of graphene, using the surface topographical corrugations like wrinkles [24,25], ripples [26,27,28,29,30,31,32,33], folds [34], or crumples [35,36,37,38] to exploit exceptional properties is an amazing technique in experiments. In particular, high-quality conformal wrinkles in graphene can be controllably fabricated with expected orientations, wavelengths, and amplitudes by using various simple methods, which is of paramount importance for potential applications in flexible electrodes, sensors, and actuators [39,40,41,42,43,44]. The wrinkling phenomenon in graphene is tied up with strain, which is one of the functionalization methods for bandgap engineering and conductivity tuning [45,46]. Tailoring graphene into nanoribbons (GNRs) is regarded as another effective approach for graphene bandgap management [47,48,49,50,51]. Encouragingly, by using self-masked plasma-etching together with a suitable transfer technique, highly aligned wrinkled GNR (WGNR) arrays can be strictly fabricated with a desirable dimension, density, and an orientation of wrinkles [52]. Recently, many studies have been carried out investigating WGNR, including sensor behavior by decorating gas molecules, the bending effect on electronic and transport properties, wrinkle orientation switching, and so forth. However, the gate-tuning effect on the charge transport property of WGNR has not been discussed, and is analyzed throughout this paper.

In the past decades, an appealing class of 1D materials emerged, named multidecker organometallic nanowires (MONWs), such as M*_n_*(Bz)*_m_* and M*_n_*(Cp)*_m_* (M = Sc − Cu, Bz = C_6_H_6_, Cp = C_5_H_5_), which have been demonstrated to have potential applicability in nano electronic and nanomagnetic devices [53,54,55]. A variety of MONWs have been successfully synthesized in experiments. A typical example is the multidecker V*_n_*(Bz)*_m_* nanowire, which exhibits extraordinary half-metallic and ferromagnetic characteristics stemmed from the spin-polarized V–V coupling [56,57]. Unlike some polymer nanowires, V*_n_*(Bz)*_m_* displays a stiff topological extension along the axial direction. In this regard, V*_n_*(Bz)*_m_* is an ideal kind of material to be positioned onto the WGNR surface to build up V_7_(Bz)_8_-WGNR vdWHs. It is anticipated that more new electrical and magnetic functionalities would be brought about aroused from the interplay between V*_n_*(Bz)*_m_* and graphene, which can be used to effectively engineer the performance of vdWHS devices. 

## 2. Models and Computation Details

### 2.1. Computational Models

**Figure 1:**
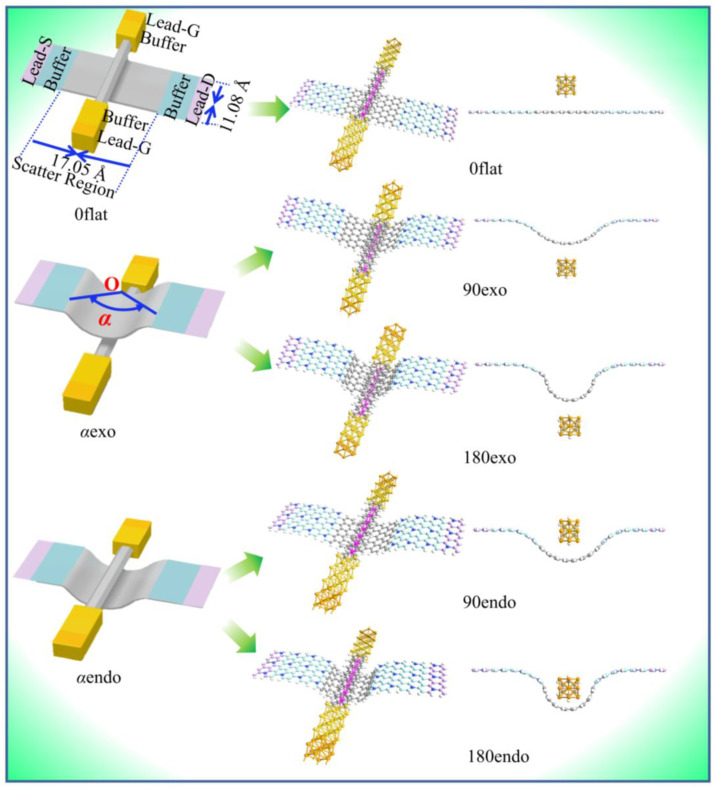
The schematic plots of the four-terminal V_7_(Bz)_8_-WGNR devices.

In this work, employing density functional theory (DFT) and nonequilibrium Green’s function (NEGF) methods, the electron tunneling behaviors with spin-polarization of five four-terminal devices with V_7_(Bz)_8_-WGNR vdWHs (Figure 1) were investigated. The calculated systems were constructed using a V_7_(Bz)_8_ (gate-to-gate channel) to cross the plane, the concave and the convex surface of WGNR (source-to-drain channel), denoted as 0flat-, endo- and exo-devices, respectively. The WGNR contained 8- and 10-layered carbon atoms along the armchair direction (*z* axle) and the zigzag direction (*x* axle), respectively. Along the *z* direction, each C atom was saturated by an H atom in order to stabilize the edge suspension bonds of WGNR. Source-to-drain and gate-to-gate separations were 42.62 and 22.24 Å, respectively, long enough to avoid lead–lead interaction. Graphene nanoribbon was the semi-conductor along the armchair direction, which was unsuitable to take as the electrode because the electrode needs to be metallic. It has been reported that doping N atoms could introduce additional π electrons to make the graphene nanoribbon become a conductor. Therefore, we adopted the N-doped graphene nanoribbon as the source lead (S-Lead) and drain lead (D-Lead) [58,59]. The Au(100)-(3×3) was selected as the gate lead (G-Lead). Three spatial curvatures of the WGNR sheet, defined by the central angle *a* = 0°, 90°, or 180°, were considered. Correspondingly, five vdWHs with V_7_(Bz)_8_-WGNR devices were designed. For the sake of description, these five systems were denoted as 0flat, 90exo, 180exo, 90endo, and 180endo, respectively, as shown in Figure 1. We found that these devices exhibit the conductance modulation of spin-dependent transport by applying different gate and bias voltages as well as via structural changes. 

### 2.2. Computational Details

The geometries of flat GNR with two N-doped GNR electrodes and V_7_Bz_8_ were optimized separately by utilizing DFT, as implemented in the Vienna ab initio simulation package (VASP). Then, the distance between the flat GNR and V_7_Bz_8_ was optimized by fixing their optimized structures. A projector-augmented wave basis set was employed with a cutoff energy of 300 eV. In the iterative calculation, the energy and force converged values were set as 10^−4^ eV and 0.05 eV·Å^−1^, respectively. To shield the interaction between the periodic images, the vacuum slab was set as 20 Å. The optimized value was 1.95 Å. Furthermore, based on the optimized geometry of the flat GNR, we constructed the WGNR structures with different curvatures. To qualitatively compare the influence of different bending curvatures on the transport properties of these four-terminal devices, the distances between WGNR and V_7_(Bz)_8_ were set as the same as that between the flat GNR and V_7_Bz_8_ (1.95 Å) for all the models. The distance between the Au electrode and V_7_Bz_8_ was also optimized by fixing their atoms positions. The optimized nearest S-Au distance is 2.34 Å, closing to their covalent bonding (2.39 Å) [60].

Transport properties of the five four-terminal conformations were calculated by using Nanodcal software package (Nanodcal 2019B) employing the DFT-NEGF method. In our simulation, the standard nonlocal norm-conserving pseudopotentials were used to describe the atomic core, while the double-zeta polarized (DZP) basis set was set for explaining the valence electronic orbitals. The exchange-correlation function was managed by the local density approximation (LDA) [61,62]. To confirm the reliability of the LDA results, we also carried out generalized gradient-approximation (GGA) calculations on the transport properties for the 0flat device at *V*_S_ = 0.0 V. The computed conductance, current, and transmission of S-D channel with the variation of *V*_G_ are shown in Figures S1–S3 in the Supporting Information. Clearly, the curves of these results exhibit a similar changing trend as varying *V*_G_ for both LDA and GGA. This means that LDA and GGA could give the same evaluation of the gate influence on S-D channel. Therefore, the use of LDA to calculate the transport properties for all the designed four-terminal systems is appropriate. The Brillouin zone was integrated using the Monkhorst–Pack method, while *k*-points were set as 1 × 1 × 100 and 100 × 1 × 1 for source-to-drain channel leads and gate channels leads, respectively, as well as that for central region was 1 × 1 × 1. Notably, the spin state of V atoms was considered in all cases. And U-J was set to 3.4 eV [56] for the LDA + U scheme, which was employed to elucidate the on-site correlation effects among the 3*d* electrons of the V atom [62]. In addition, the energy and force convergence tolerance of self-consistent calculations were set as less than 10^−4^ eV and 0.05 eV/Å, respectively. 

The spin-dependent current of the *α*-lead in a multi-terminal system can be evaluated using the Landauer–Büttiker formula [63,64]:

(1) $$I_{\sigma }=\frac{e}{h}\sum_{\beta }\int_{-\infty }^{+\infty }dET_{\alpha \beta }\left(E,V_{\alpha },V_{\beta }\right)\left[f_{\beta }\left(E\right)-f_{\alpha }\left(E\right)\right]$$

where *V_α_* (*V_β_*) and *f_α_* (*f_β_*) denote the electron potentials and the Fermi distribution functions of the *α*-lead (*β*-lead), respectively. *T_αβ_* presents the transmission coefficient between *α*-and *β*-leads. *σ* is the spin index, ↑ (up-spin) and ↓ (down-spin). The total current is the sum of *I*_↑_ and *I*_↓_.

The conductance *G_αβ_* is related to *T_αβ_*, which can be obtained by the following formula:

(2) $$G_{\alpha \beta }=\frac{e^{2}}{h}T_{\alpha \beta }\left(E\right)$$

## 3. Results and Discussions

The configurations of vdWHs devices of 0flat, 90exo, 180exo, 90endo, and 180endo are presented in Figure 1. In the forthcoming presentations, the gate-tuned transport properties of V_7_(Bz)_8_-WGNR devices were detected by evaluating the conductance, spin currents, transmission, local density of states, and scattering states. The source voltages (*V*_S_) were chosen as 0.0, 0.2, and 0.4 V. At a given *V*_s_, the gate voltages (*V*_G_) were selected as 0.0, 0.2, 0.6, and 1.0 V. 

**Figure 2:**
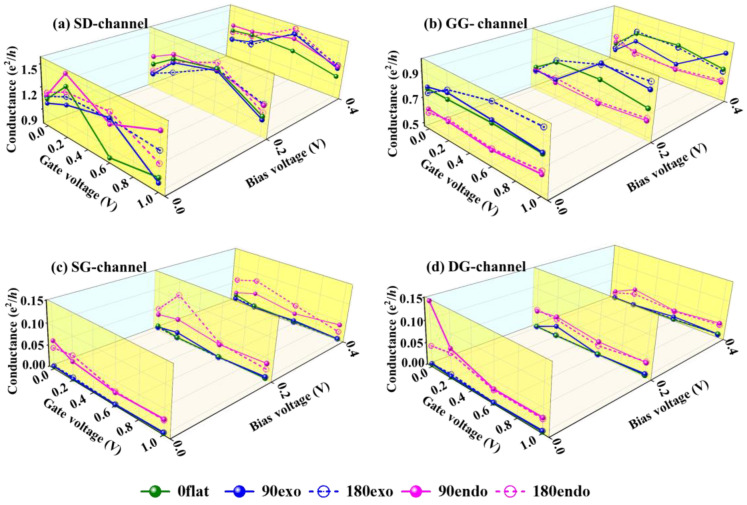
Conductance of (**a**) SD-, (**b**) GG-, (**c**) SG-, and (**d**) DG-channels with various *V*_G_ at a constant *V*_S_ = 0.0, 0.2, or 0.4 V in the four-terminal V_7_(Bz)_8_-WGNR devices.

The conductance as a function of *V*_G_ at a fixed *V*_S_ (*V*_S_ = 0.0, 0.2, and 0.4 V) of all channels in the five four-terminal devices is shown in Figure 2. The conductance was in the range of 0.9–1.5 e^2^/h for the SD-channel, while it went down to 0.5–0.9 e^2^/h for the GG-channel. Such a difference derives from the fact that graphene possesses a metallic characteristic while V_7_(Bz)_8_ has a half-metallic feature. The conductance for SG- and DG-channels is always below 0.15 e^2^/h. The V_7_(Bz)_8_ attaches to graphene merely via a vdW interaction, so an unstraight way for carrier transporting along SG- and DG-channels is generated, leading the weaker conductivity. It is worth noting that, in the 0flat device, the conductance for SG- and DG-channels is almost zero in all cases, indicating that the channels between V_7_(Bz)_8_ and graphene are nearly closed. Clearly, the conductance does not always obey Ohm’s law, it sometimes oscillates with the variation of *V*_G_. Overall, with the growing *V*_G_, the conductance magnitude of SG-, and DG-channels is suppressed because of the stronger confrontation of gate potential against source potential. Simultaneously, the conductance of the SD-channel always goes up and down. Based on these results, it can be concluded that the change of *V*_G_ can strongly influence the resistance in these channels, i.e., the precise regulation of conductance can be realized by varying *V*_G_.

Furthermore, the conductance of SD-, SG-, and DG-channels in an endo-device is larger than that of the corresponding exo-device (pink lines vs. blue lines of Figure 2), especially that of SG-, and DG-channels. The gate and source potentials couple stronger in endo-devices, owing to the larger contact area between V_7_(Bz)_8_ and graphene. Accordingly, a counteractive effect is observed for GG-channel; that is, the conductance is smaller in endo-devices than that in exo-devices. Therefore, different position of V_7_(Bz)_8_ can bring about different coupling between V_7_(Bz)_8_ and WGNR. Meanwhile, the bending curvature also impacts the electronic properties as it can induce localization or delocalization in the electron state. This change in the charge distribution may result in modifications of conductance. Evidently, the bending curvature has a slight effect on the conductance of endo- and exo-devices which makes the values go a little up or down, depending on the complex potential couplings between these channels as well as their intrinsic electronic properties.

**Figure 3:**
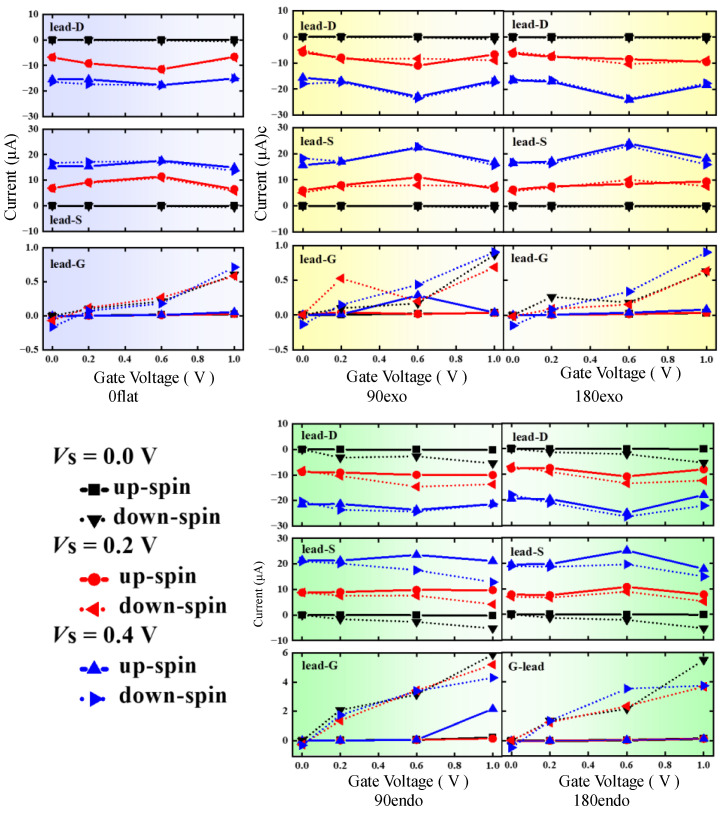
Spin currents of S-, D-, and G-leads of the five four-terminal V_7_(Bz)_8_-WGNR devices with varying *V*_G_ at a constant *V*_S_ = 0.0, 0.2, and 0.4 V.

It is well known that V_7_(Bz)_8_ is a half-metallic conductor which is dominated solely by the down-spin channel. The up-spin and down-spin currents of each lead in the five four-terminal devices were computed to examine the gate-tuning effect of V_7_(Bz)_8_ on the transport property. For simplicity, the symbol *I*_S↑_ and *I*_S↓_ are used to signify the up- and down-spin current crossing the S-lead, respectively, while *I*_D↑_ and *I*_D↓_ as well as *I*_G↑_ and *I*_G↓_ are employed for those of the D-lead, and the G-lead, respectively. These values are given in Figure 3 varying with *V*_G_ at each fixed *V*_S_. The value of the current flowing from the lead to the center region is positive, named input current, and that of the opposite direction is negative called output current. The colors of the current curves are black, red, and blue for *V*_S_ = 0.0, 0.2, and 0.4V, respectively. Solid and dash lines are for up-spin and down-spin pathways, respectively. By comparing the curve shape of Figure 2 and Figure 3, it is readily observable that the shapes of S-lead and D-lead current curves are much more similar to the conductance curve of the SD-channel, indicating that the currents of S- and D-leads are stronger dependent on the current passing SD-channel. Actually, the current of each lead is always the sum of these from all its related channels. In addition, more complicated transport characteristics may be aroused by the mutual couplings between leads owing to the multi-terminal setup. 

As anticipated, in V_7_(Bz)_8_, the magnitude of *I*_G↓_ is much higher than that of *I*_G↑_ originated from its native half-metallic behavior. However, such spin-polarized character has no significant effect on the electronic properties of 0flat, 90exo, and 180exo devices. This is due to the fact that the current of G-leads is an order of magnitude lower than that of S- and D-leads, i.e., the contact between V_7_(Bz)_8_ and WGNR in these systems is insufficient to induce noticeable perturbations by the applied gate voltages on the graphene main channel. However, the situation is different for 90endo and 180endo devices, where the polarized characteristic of V_7_(Bz)_8_ is almost completely imposed on the source-to-drain pathway, inducing striking polarized currents of S- and D-leads as well. Thus, the analysis mainly focuses on 90endo and 180endo devices in the following discussions.

Upon applying a higher *V*_G_, the currents across S- and D-leads are polarized to a larger extent. At *V*_S_ = 0.0 V, S-lead exports only down-spin *I*_S↓_ which is driven by the spin-polarized gate voltage. Once *V*_S_ is applied, currents *I*_S↑_ and *I*_S↓_ are both input currents while *I*_D↑_ and *I*_D↓_ are output currents. The curves of up-spin *I*_D↑_ and *I*_S↑_ are almost unchanged with the variation of the *V*_G_ at a certain *V*_S_, which is mainly owing that *V*_G_ and *V*_S_ cannot activate the up-spin channel of V_7_(Bz)_8_ evidently in these situations, leading to scarcely any disturbance on the up-spin channel of WGNR. 

Strong coupling occurs between the down-spin channels of V_7_(Bz)_8_ and WGNR, resulting in the splitting of up- and down-currents (see Figure 3). Evidently, the input *I*_S↓_ is always smaller than *I*_S↑_, while the output *I*_D↓_ is larger than *I*_D↑_. These results mean that the V_7_(Bz)_8_ tunes the spin-polarized transport properties of WGNR by importing the down-spin state carriers. The current *I*_G↓_ coming from V_7_(Bz)_8_ suppresses the input *I*_S↓_ leading to a smaller value compared with *I*_S↑_. On the contrary, down-spin current from V_7_(Bz)_8_ superimposes on the output magnitude of *I*_D↓_, and consequently, the spin-polarized current is generated by giving a larger *I*_D↓_ than *I*_D↑_. Such spin polarization becomes more obvious with the increasing of *V*_G_. The 90endo device produces a bit more evident splitting of the up- and down-current than the 180endo device. The bending curvature may be responsible for these phenomena: WGNR gives a smoother pathway in 90endo than in 180endo.

**Figure 4:**
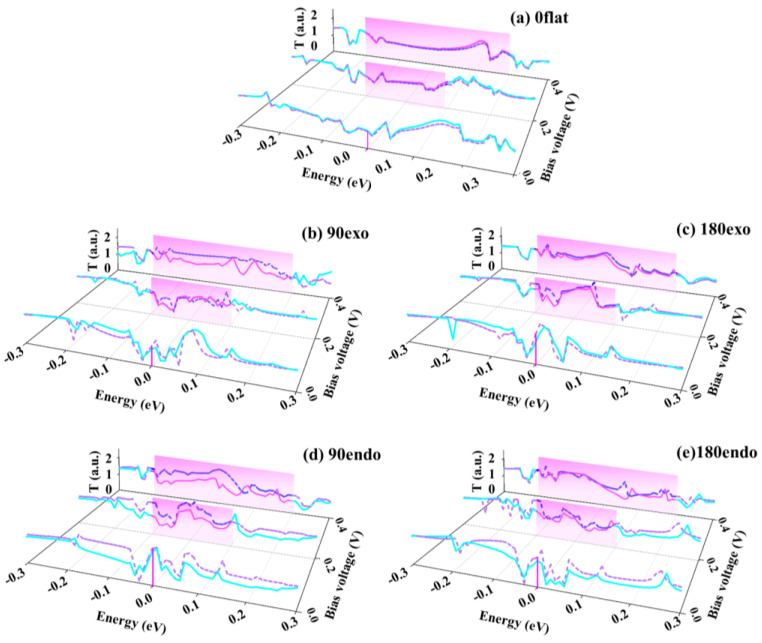
Transmission spectra of the four-terminal V_7_(Bz)_8_-WGNR devices at *V*_G_ = 1.0 V, where (**a**) is the TS for 0flat device, (**b**), (**c**), (**d**) and (**e**) are that for 90exo, 180exo, 90endo, 180endo devices, respectively. The solid lines represent the TS of the up-spin state, the dash lines that for the down-spin state. The magenta and bule lines denote the TS of up-spin and down-spin states in the bias window, respectively. The pink regions indicate the bias window.

To make the transport property of the four-terminal V_7_(Bz)_8_-WGNR device more transparent, the transmission spectra (TS), which is dependent on the energy, was computed for the SD-channel. One can easily find evidence that the TS is closely related to the current based on the Landauer–Bütiker formula. Generally, the integral area of T (*E*, *V*) in the bias windows determines the current, where the bias window is referred to [−*V*/2, *V*/2] when the Fermi level is set to 0.0 eV. And the larger integral area exists, the higher current is. Figure 4 plots their TS at each applied *V*_S_ when *V*_G_ is fixed at 1.0 V for all the devices, where the pink region represents the bias window. For the 0flat device, there is almost no spin splitting in TS by tuning *V*_S_ as shown in Figure 4a. However, by careful observation, one can find that the down-spin channels of exo- and endo-devices have a larger TS integral area in the bias window than the up-spin channels, and the spin splitting of 90exo and 90endo devices is more apparent than that of 180exo and 180endo devices. These results further confirm that the V_7_(Bz)_8_ can tune the down-spin transport properties of WGNR prominently by various *V*_G_. At the same time, the bending curvature and the position of V_7_(Bz)_8_ can also influence the down-spin state transport of WGNR.

The distributions of the local density of states (LDOS) can also reveal the transport property of the four-terminal V_7_(Bz)_8_-WGNR devices. The diagrams of LDOS with various *V*_S_ (0.0, 0.2, and 0.4V) at *V*_G_ = 1.0 V are presented in Table 1. The change in the color of LDOS can give a sign of the change in the electronic potential distribution. Through comparative analyses of these figures, one can easily find that the distribution of the down-spin state LDOS of V_7_(Bz)_8_ is much more delocalized than that of the up-spin state. The down-spin channel of WGNR maybe perturbed by V_7_(Bz)_8_, which gives the reason to make a spin-polarized behavior of these four-terminal devices. Evidently, the *V*_S_, the bending curvature of WGNR, and the position of V_7_(Bz)_8_ all can tune the LDOS distribution of the whole system. The brighter LDOS color in the endo-devices than that in the exo-devices and 0flat device indicates that V_7_(Bz)_8_ has a stronger disturbance to WGNR in these cases, which explains why the polarized character is more outstanding in endo-devices in Figure 3. The scattering states of S- and D-leads in the four-terminal devices at *V*_G_ = 1.0 V are summarized in Table 2 to further make the polarized transport character more patently. The charge carries through the up-spin channel are scattered stronger than that across the down-spin pathway. The polarized transport properties of WGNR are mainly induced by the tuning of the down-spin state of V_7_(Bz)_8_. It is worth noting that both the bending curvature of WGNR and the position of V_7_(Bz)_8_ could influence the polarized effect, especially, the 90endo-device shows the most obvious polarized characteristic.

## 4. Conclusions

Based on the DFT-NEGF method, the transport property of four-terminal V_7_(Bz)_8_-WGNR devices tuned by the polarized gate were investigated by analyzing the conductance, spin-polarized current, TS, LDOS, and scattering states. The results show that the position of V_7_(Bz)_8_, as well as the bending curvature of WGNR, can exert important influences on the transport property. The tuning effect of V_7_(Bz)_8_ mainly depends on its down-spin state, which can induce a spin-polarized transport property for these four-terminal devices. Particularly, the down-spin channel disturbs strongly on the source-to-drain conductance of WGNR when V_7_(Bz)_8_ is endo-positioned crossing the WGNR. We trust that these results could give guidance for designing flexible graphene-based electronic nanodevices by attaching other similar multidecker metal-arene nanowires.

## Figures and Tables

**Table 1:** Local density of states of the four-terminal V_7_(Bz)_8_-WGNR devices at various *V*_S_ exemplified by *V*_G_ = 1.0 V.

Model	Spin State	0.0 V	0.2 V	0.4 V
0	Up-spin	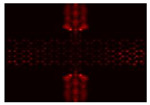	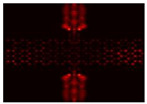	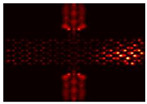
	Down-spin	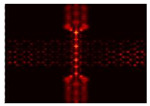	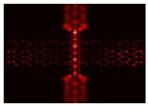	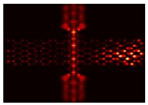
90exo	Up-spin	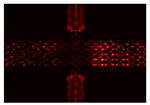	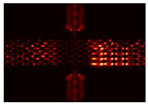	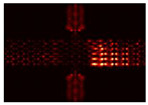
	Down-spin	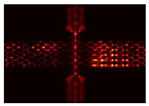	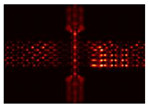	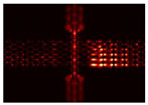
180exo	Up-spin	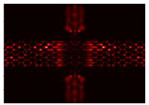	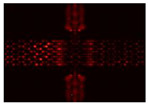	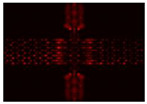
	Down-spin	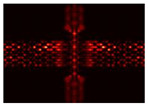	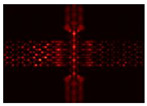	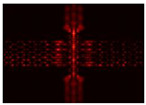
90endo	Up-spin	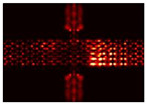	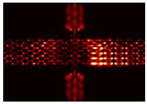	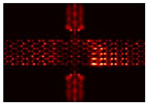
	Down-spin	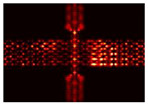	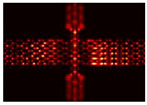	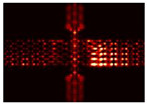
180endo	Up-spin	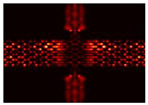	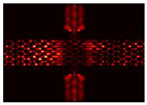	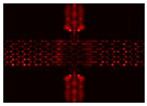
	Down-spin	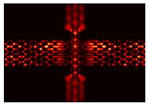	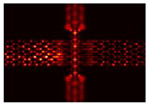	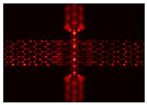

**Table 2:** Scattering states of S-lead and D-lead at various *V*_S_ in the four-terminal V_7_(Bz)_8_-WGNR devices exemplified by *V*_G_ = 1.0 V.

Model	*V*_S_	S-Lead		D-Lead	
		Up-Spin	Down-Spin	Up-Spin	Down-Spin
**0**	0.0 V	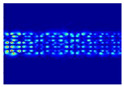	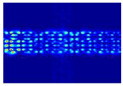	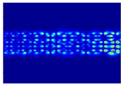	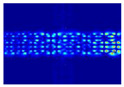
	0.2 V	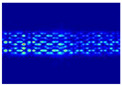	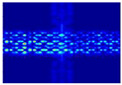	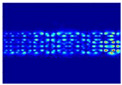	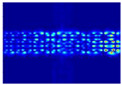
	0.4 V	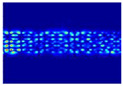	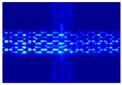	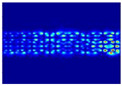	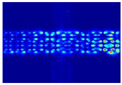
90exo	0.0 V	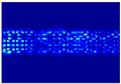	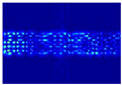	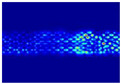	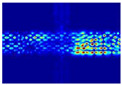
	0.2 V	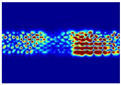	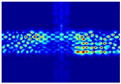	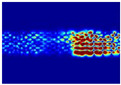	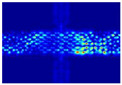
	0.4 V	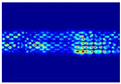	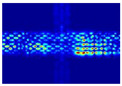	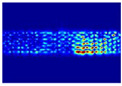	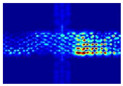
180exo	0.0 V	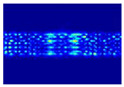	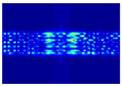	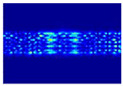	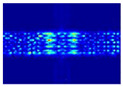
	0.2 V	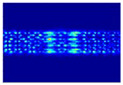	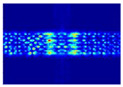	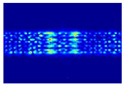	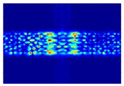
	0.4 V	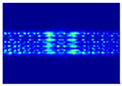	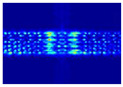	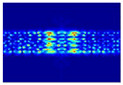	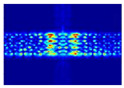
90endo	0.0 V	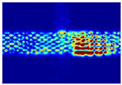	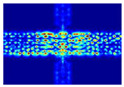	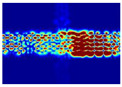	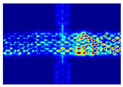
	0.2 V	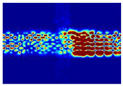	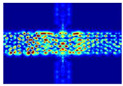	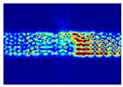	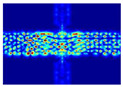
	0.4 V	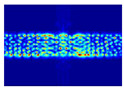	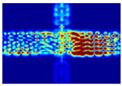	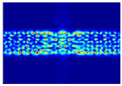	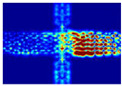
180 endo	0.0 V	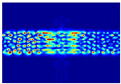	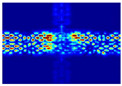	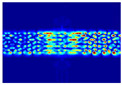	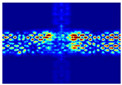
	0.2 V	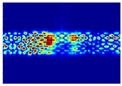	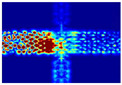	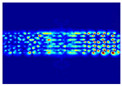	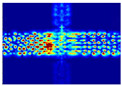
	0.4 V	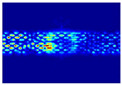	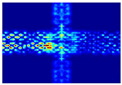	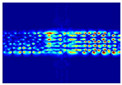	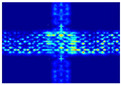

## Data Availability

The data presented in this study are available on request from the corresponding authors.

## Supplementary Materials

The following supporting information can be downloaded at: https://www.mdpi.com/article/10.3390/nano13152270/s1, Figure S1: Comparison of LDA and GGA calculated conductance of S-D channel at *V*_S_ = 0.0 V for the 0flat device; Figure S2: Comparison of LDA and GGA calculated currents of S-lead and D-lead at *V*_S_ = 0.0 V in the 0flat device; Figure S3: Comparison of LDA and GGA calculated transmission coefficient of S-D channel at *V*_S_ = 0.0 V in the 0flat device.
