# Supplementary material for: Transport Property of Wrinkled Graphene Nanoribbon Tuned by Spin-Polarized Gate Made of Vanadium-Benzene Nanowire
Source: Nanomaterials (Basel). 2023 Aug 7;13(15):2270. doi: 10.3390/nano13152270 (PMC10421401; doi:10.3390/nano13152270)
Supplement: Supplementary file 1 [file nanomaterials-13-02270-s001.zip › nanomaterials-2436218-supplementary.pdf]

Supporting Information for

Transport Property of Wrinkle Graphene Nanoribbon Tuned by Spin-Polarized Gate Made of $V_7(Bz)_8$ Nanowire

Hong Yu, Yan Shang*, Lei Pei, Yangyang Hu, Guiling Zhang*

School of Materials Science and Chemical Engineering, Harbin University of Science and Technology, Harbin 150080, China.

**Corresponding author (email: guiling-002@163.com; shangyan1972@126.com)*

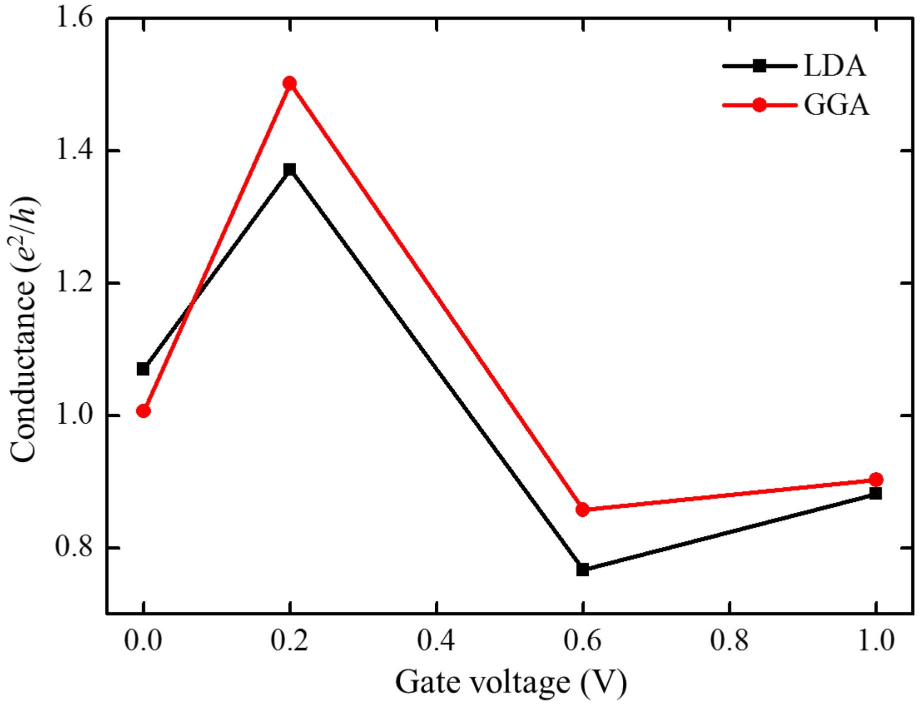

Figure S1 Comparison of LDA and GGA calculated conductance of S-D channel at V_S = 0.0 V for the 0flat device.

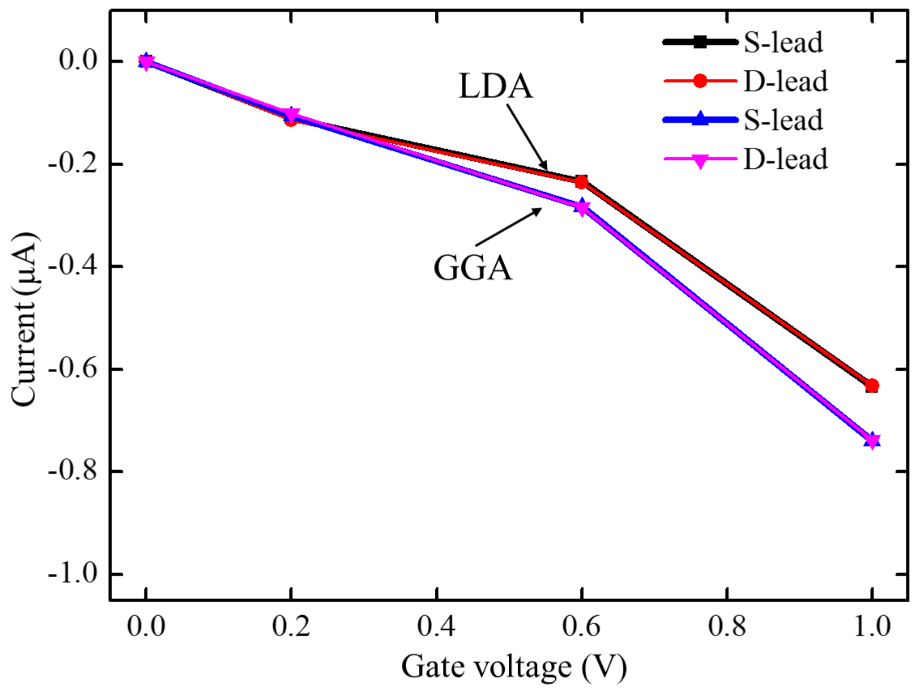

Figure S2 Comparison of LDA and GGA calculated currents of S-lead and D-lead at V_S = 0.0 V in the 0flat device.

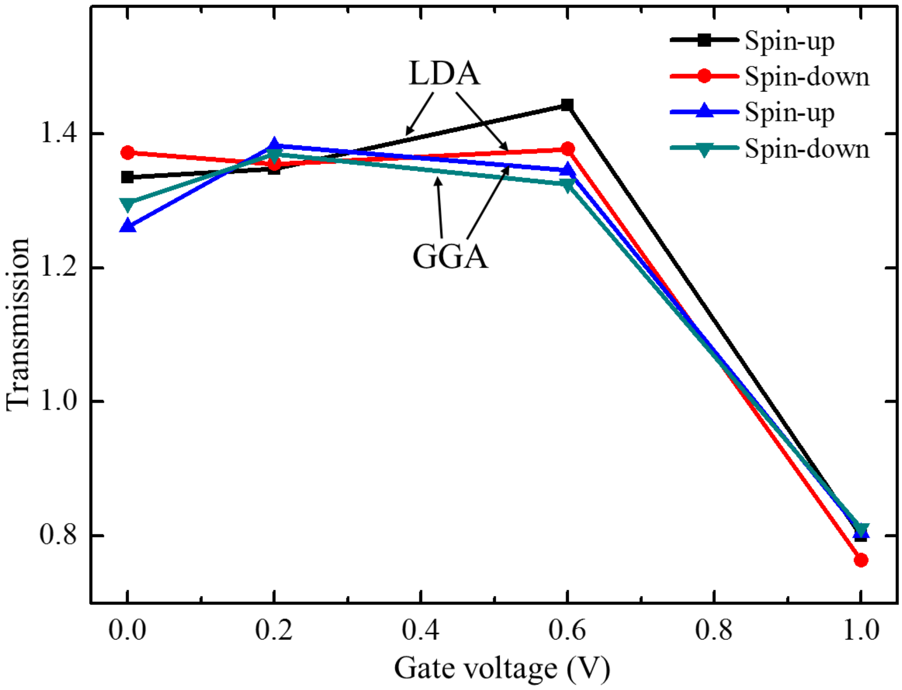

Figure S3 Comparison of LDA and GGA calculated transmission coefficient of S-D channel at V_S = 0.0 V in the 0flat device.
